# Molecular Evolution of Severe Acute Respiratory Syndrome Coronavirus 2: Hazardous and More Hazardous Strains Behind the Coronavirus Disease 2019 Pandemic and Their Targeting by Drugs and Vaccines

**DOI:** 10.3389/fcimb.2021.763687

**Published:** 2021-12-14

**Authors:** Hardeep Singh Tuli, Katrin Sak, Poonam Aggarwal, Ashif Iqubal, Sushil K. Upadhaya, Jagjit Kaur, Ginpreet Kaur, Diwakar Aggarwal

**Affiliations:** ^1^ Department of Biotechnology, Maharishi Markandeshwar (Deemed to be University), Mullana, India; ^2^ Non-Governmental Organization (NGO) Praeventio, Tartu, Estonia; ^3^ The Basic Research Laboratory, Center for Cancer Research, National Cancer Institute at Frederick, National Institutes of Health, Frederick, MD, United States; ^4^ Department of Pharmacology, School of Pharmaceutical Education and Research (Formerly Faculty of Pharmacy), Jamia Hamdard (Deemed to be University), Delhi, India; ^5^ Graduate School of Biomedical Engineering, ARC Centre of Excellence in Nanoscale BioPhotonics (CNBP), Faculty of Engineering, The University of New South Wales, Sydney, NSW, Australia; ^6^ Shobhaben Pratapbhai Patel School of Pharmacy and Technology Management, Shri Vile Parle Kelavani Mandal, Narsee Monjee Institute of Management Studies (SVKM’S NMIMS), Mumbai, India

**Keywords:** COVID pandemic, variants, molecular evolution, therapeutics, vaccination

## Abstract

Within almost the last 2 years, the world has been shaken by the coronavirus disease 2019 (COVID-19) pandemic, which has affected the lives of all people. With nearly 4.92 million deaths by October 19, 2021, and serious health damages in millions of people, COVID-19 has been the most serious global challenge after the Second World War. Besides lost lives and long-term health problems, devastating impact on economics, education, and culture will probably leave a lasting impression on the future. Therefore, the actual extent of losses will become obvious only after years. Moreover, despite the availability of different vaccines and vaccination programs, it is still impossible to forecast what the next steps of the virus are or how near we are to the end of the pandemic. In this article, the route of molecular evolution of the coronavirus severe acute respiratory syndrome coronavirus 2 (SARS-CoV-2) is thoroughly compiled, highlighting the changes that the virus has undergone during the last 2 years and discussing the approaches that the medical community has undertaken in the fight against virus-induced damages.

## Introduction

From the end of 2019, life has been greatly affected by the coronavirus disease 2019 (COVID-19) all over the world. Based on the data from Worldometers, this pandemic has afflicted more than 241.97 million human lives and has claimed nearly 4.92 million lives around the globe during the last 1.5 years (https://www.worldometers.info/coronavirus/; data from October 19, 2021). At that, the elderly people and those with underlying cardiovascular, respiratory, and metabolic disorders have been found to be especially vulnerable by severe course of the disease, causing bilateral pneumonia, acute respiratory distress syndrome (ARDS), failure of multiple organs (including, but not limited to, the brain, heart, liver, and kidneys), or even mortality (Abdullahi et al., 2020; Li et al., 2021). In addition to the direct health damages, devastating impact on education, culture, economics, and general public welfare proceeding from the strict restrictions in social contacts established for the disease prevention cannot be underestimated (Sood et al., 2020).

COVID-19 is caused by an infection with the single-stranded RNA virus with positive polarity, i.e., severe acute respiratory syndrome coronavirus 2 (SARS-CoV-2) that transmits mainly *via* respiratory droplets, aerosols, and fomites (Abdullahi et al., 2020; Kawabata et al., 2020; Li et al., 2021; Mallah et al., 2021). Coronaviruses consist of enveloped virus particles with 80–120 nm of diameter; they have typically spherical or pleomorphic structure with spike-like projections of glycoproteins on surface, giving them a crown-like appearance under electron microscopy (Tuli et al., 2021). The initial reservoir of SARS-CoV-2 is hypothesized to be bats transmitting the virus particles to human beings (Tuli et al., 2021). Within the time of the pandemic course, SARS-CoV-2 virus has been in a continuous molecular evolution, displaying genetic diversity and mutations with varied degrees of transmission and virulence (Abdullahi et al., 2020; Deimel et al., 2021). Such mutations can help virus particles to escape the immune system and/or replicate more efficiently once it has entered the host organism, making the virus more infectious and pathogenic (Adedokun et al., 2021; Hossain et al., 2021). The impact of viral changes on the COVID-19 pandemic has been apparent in the disease outbreaks occurring disproportionately in different parts of the world (Abdullahi et al., 2020; Fraser, 2020; Vudathaneni et al., 2021). Therefore, the virus variants are designated by the geographical regions where the mutations have emerged, including the UK (B.1.1.7), Brazilian (B.1.1.248), and South African (1.351) strains, among others (Hossain et al., 2021). Furthermore, as mutations in the virus genome can change also the susceptibility of the virus to both clinically used drugs and vaccines, concerns have been arisen about the efficacy of current preventive and therapeutic interventions for stopping the pandemic (Chiam et al., 2021; Hossain et al., 2021; Matta et al., 2021; Robinson et al., 2021).

In this state-of-the-art review article, molecular characteristics of the currently emerged variants of SARS-CoV-2 are under discussion, analyzing their infectivity, morbidity, and mortality potential, as well as susceptibility to the current intervention measures applied for achieving control over the pandemic.

## Molecular Evolution of Coronavirus Disease 2019 From Its Emergence to the Current State

Mutations originate as a result of viral replication during circulation. Despite being an RNA virus, coronaviruses undergo fewer mutations because of their strong proofread mechanism. Moreover, the fate of mutations is determined by the natural selection, meaning that those favored with respect to viral better survival will increase in frequency, and those that reduce viral fitness tend to be eliminated from the population of circulating viruses. However, mutations can also happen due to chance events. Therefore, the interplay of natural selection and chance events leads to virus evolution.

The SARS-CoV-2 virus has been mutated over time, resulting in different genetic variations in the population of circulating viral strains over the course of the COVID-19 pandemic. The evolution of SARS-CoV-2 suggests strong purifying selection and modest divergence; one of the most closely related strain of SARS-CoV-2 is “RaTG13” found in a bat sample from Yunnan Province, China, in 2013. RaTG13 (horseshoe bat, *Rhinolophus affinis*) shows 96% similarity to SARS-CoV-215. Though RaTG13 is closely related to SARS-CoV-2, there is a significant level of variation in sequence similarity across the genomes of these two viruses, ranging between 93.1% and 99.6% (Zhou et al., 2020). However, comparisons with other coronavirus strains suggest complex recombination events during its evolution. Various recombinations were detected across the genome majorly in ORF1a and in the region marking the N-terminus of the S protein (Li et al., 2003; Li, 2016; Hoffmann et al., 2020; Wan et al., 2020). S protein binds to angiotensin-converting enzyme 2 (ACE2) receptors and mediates viral entry into the human cells. One such mutation, D614G, arises as a result of single-nucleotide polymorphism (SNP) and results in amino acid change from an aspartate [D] to a glycine [G] at residue 614, increasing the efficiency of viral entry into the human cells (Isabel et al., 2020; Korber et al., 2020).

The D614G mutation in the spike glycoprotein of SARS-CoV-2 was significantly detected for the first time in early March 2020 and has spread globally across multiple geographic regions over the next month (Korber et al., 2020). However, various sequencing studies have already identified the D614G mutation in viruses in China in late January, which dispersed globally. Similarly, the population genetics analysis of more than 25,000 sequences from the United Kingdom also found that viruses with 614G are more transmissible and affect larger phylogenetic clusters (Volz et al., 2021). Even parallel studies in animal models also indicate that 614G viruses are more transmissible. As a result of more favored mutation, this strain has now become a dominant global strain (Hou et al., 2020; Plante et al., 2021).

Apart from its evolution in humans, there is evidence of cross-specific transmission in other animals like mink, which can even lead to emergence of potentially dangerous recombinant SARS-CoV-2 strains. Outbreaks of SARS-CoV-2 on mink farms in the Netherlands and Denmark that started in late spring and early summer 2020 demonstrated human-to-mink, mink-to-mink, and mink-to-human transmissions (European Centre for Disease Prevention and Control, 2020, Oude Munnink et al., 2021). In early November 2020, 214 cases of mink-associated human COVID-19 were reported. These cases where Y453F mutation in the receptor binding domain of spike might be responsible for increased binding affinity for ACE2 in mink. Eleven patients from the Danish outbreak had a cluster 5 variant having three additional mutations in spike (del69_70, I692V, and M1229I). An investigation of human serum samples in nine patients showed a significant reduction in neutralization activity against cluster 5 viruses (mean, 3.58-fold; range, 0–13.5). Therefore, continued evolution and adaptation of SARS-CoV-2 in an animal reservoir resulted in novel SARS-CoV-2 from mink to humans and other mammals.

Another lineage B.1.1.7 (also called 501Y.V1) was identified in southeastern England (Rambaut et al., 2021) and became one of the variants of the highest concern. This variant has already highly evolved, having 17 lineage-defining mutations even prior to its detection in early September. Seven of these mutations were in the spike proteins only that later formed the basis for the vaccine in the United Kingdom. This variant was found to be 56% more transmissible and was responsible for approximately 28% of cases of SARS-CoV-2 infection in England within 1 month (Davies et al., 2020). Unlike D614G, which could be because of chance events, B.1.1.7 (Alpha variant) strongly seems to have arisen as a result of natural selection. It came into existence after outcompeting already circulating widespread SARS-CoV variants.

Most of the mutations in B.1.1.7 lineage include mutations in the spike glycoprotein, N501Y in the receptor binding domain, deletion 69_70, and P681H in the furin cleavage site, which could probably influence ACE2 binding and viral replication. Specifically, the 501Y spike variants were predicted to have an increased affinity for human ACE2, and another variant, also with an N501Y mutation, was spreading fast in South Africa (Beta variant—B.a351, B.1.351.2, and B.1.353.3). Immunogenic effects of these mutations are currently not clear. Similarly, the Gamma variant (P.1) was emerged in the Amazon city of Manaus in December 2020 and has led to a surge in cases in Brazil (Buss et al., 2021).

Recently, the Delta variant (B.1.617.2, AY.1, and AY.2) having multiple mutations originated in India is of major concern (Centers for Disease Control and Prevention (CDC), 2021; Public Health England, 2021). This variant is the highest transmissible variant and hence favored by evolution. Therefore, different mutants originated in different geographical areas as a combinatorial result of selective advantage or chance mutation. Variants having mutations in spike to increase transmissibility could quickly outcompete and replace other circulating variants. Moreover, widespread infection among humans is now posing a huge threat to other mammals that usually interact with human populations and worsen the severity of disease by creating more dangerous recombinant SARS-CoV-2 strains. It would be important to consider the epidemiological, genetic, and functional studies of different variants and come up with a strong strategy to stop its transmission across the species.

## Geographically Emerged Strains and Their Structural Differences

Accumulation of mutations within the genome is the primary driving force in viral evolution within an endemic setting (Dan et al., 2020; Baden et al., 2021). This inherent feature often leads to altered virulence, infectivity and transmissibility, and antigenic shifts to escape host immunity, which might compromise the efficacy of vaccines and antiviral drugs (Upadhyay et al., 2021; Yadav et al., 2021a). The SARS-CoV-2 as RNA virus lacks mismatch repair mechanism and replication accompanied by a high mutation rate (Domingo and Holland, 1997). Therefore, the mutations of the coronavirus are commonsensical and predictable, which leads to several rapidly spreading variants (**Table 1**). At present, emergence of fast-spreading three SARS-CoV-2 variants (B.1.1.7, B.1.351, and B.1.1.28.1) due to rapid mutations in ACE2 became dominant strains all around the world, causing concern on prevention and treatment of COVID-19 (Krammer, 2020; Callaway, 2021; Zhou and Wang, 2021). The morphological and physiological assessments of the P.1 or B.1.1.28.1 variant of SARS-CoV-2 from Brazil reflected less resistance to antibodies produced from natural infection or vaccination compared with other parallel variants B.1.351 from South Africa, and B.1.1.7 from the United Kingdom (Faria et al., 2021). It is noteworthy that P.1, B.1.1.7, and B.1.351 have accrued multiple mutations in the NTD (N-terminal domain) and can be neutralized by a monoclonal antibody, mAb 222 (Cerutti et al., 2021; Dejnirattisai et al., 2021). In addition, these mutated residues also have the potential to modulate vaccine-induced antibody responses (Supasa et al., 2021; Zhou et al., 2021). The three central variants by analyzing 160 sequences claimed that B-type viruses (with substitution, NS8_L84S) were common in East Asia, whereas A-type (ancestral lineage) and C-type (NS3_G251V variant) viruses were prevalent in Europe and North America (Forster et al., 2020). Along with other co-evolving mutations, NSP12_P323L and S_D614G probably provide variants with an evolutionary advantage over their ancestral types, allowing them to survive and circulate in this densely populated region (Becerra-Flores and Cardozo, 2020; Islam et al., 2021). Thus, the recent emergence of a number of variants of concern (VOCs) has led to design of new vaccines that will be able to protect against the emerging viral variants.

**Table 1:** Different variants of SARS-CoV-2 according to the WHO.

S. no.	Variant name	1st detected by	Month, year of detection	Key mutations in spike protein	Reference
**1**	614G	Bavaria, Germany	January, 2020	D614G	Brüssow, 2021; Plante et al., 2021
**2**	20C-US	United States	May, 2020	Q677; Q173	Pater et al., 2021
**3**	B.1.427/B.1.429 (also known as Epsilon variant)	United States	June, 2020	L452R; W152C; S13I; D614G	Tomkins-Tinch et al., 2021
**4**	B.1.1.7 (also known as 20I/501Y.V1 or VOC202012/01 or Alpha variant)	United Kingdom	September, 2020	H69/V70; Y144; N501Y; A570D; P681H	Leung et al., 2021; Sarkar et al., 2021a
**5**	CAL., 20C	Southern California	October, 2020	ORF1a: I4205V; ORF1b: D1183Y; S13I; W152C; L452R	Zhang et al., 2021
**6**	B.1.526 (also known as Iota variant)	United States	November, 2020	L5F; T95I; D253G; D614G; A701V; E484K or S477N	West et al., 2021b
**7**	B.1.525 (also known as Eta variant)	United Kingdom, Nigeria	December, 2020	H69-V70; Y144; Q52R; E484K; Q677H; D614G; F888L	Faria et al., 2021
**8**	B.1.351 (also known as 20H/501Y.V2 or Beta variant)	South Africa	December, 2020	L242/A243/L244; K417N; E484K; N501Y	Tegally et al., 2021; WHO, 2021a
**9**	B.1 descendant with 9 mutations	France	January, 2021	G142; D66H; Y144V; D215G; V483A; D614G; H655Y; G669S; Q949R; N1187D	West et al., 2021a
**10**	B.1.1.28.1 (also known as P.1 or Gamma variant)	Brazil/Japan	January, 2021	K417T, E484K; N501Y	Sabino et al., 2021; Chudik et al., 2021
**11**	B.1.1.28.3 (also known as P.3 or Theta variant)	Philippines	February, 2021	E484K; N501Y; P681H	Haseltine, 2021
**12**	B.1.1.28.2 (also known as P.2 or Zeta variant)	Brazil	April, 2021	L18F; T20N; P26S; F157L; E484K; D614G; S929I; V1176F	Faria et al., 2021
**13**	B.1.617.2 (also known as Delta variant)	London, United Kingdom, India	March–May, 2021	T19R, (V70F*), T95I, G142D, E156-, F157-, R158G, (A222V*), (W258L*), (K417N*), L452R, T478K, D614G, P681R, D950N	Salvatore et al., 2021; Williams et al., 2021
**14**	B.1.617.1/B.1.617.3 (also known as Kappa variant)	Maharashtra India	February, 2021	G142D; E154K; L452R; E484Q; D614G; P681R; Q1071H	Cherian et al., 2021; WHO, 2021b

SARS-CoV-2, severe acute respiratory syndrome coronavirus 2.

The comprehensive analysis of whole-genome sequences of 837 Indian SARS-CoV-2 strains revealed the occurrence of 33 different mutations, 18 of which were unique to India (Tang et al., 2020; Sarkar et al., 2021b). The second SARS-CoV-2 epidemic wave in India began around March 2021, and just weeks after, it became the dominant lineage by superseding the previous lineages (Kar et al., 2021; Salvatore et al., 2021). Almost all new cases of COVID-19 are the Delta variant (B.1.617.2) with augmented cases, but the rate of growth is slower than that of the Alpha variant (O’Dowd, 2021). The data showed the even at the higher risk of hospitalization for patients with the Delta variant compared with the Alpha variant (B.1.1.7), two doses of vaccine gave a high degree (90%) of protection (Shrotri et al., 2021; Stowe et al., 2021; Williams et al., 2021). The identification and spread of various dreading variants including B.1.1.7, B.1.351, and P.1 in India led to global VOCs (Alai et al., 2021). The Kappa and Delta variant lineages of SARS-CoV-2 were first detected in December 2020 in India (Cherian et al., 2021). Rapidly between January and February 2021, the Delta (B.1.617.2) variant became dominant in Maharashtra and was marked as a VOC in early May by the WHO (2021b). Therefore, it is imperative that currently known variants of COVID-19 and new variants should be carefully considered in the design of an effective vaccine.

### Variability in Infectivity, Morbidity, and Mortality of Different Strains

**Figure 1:**
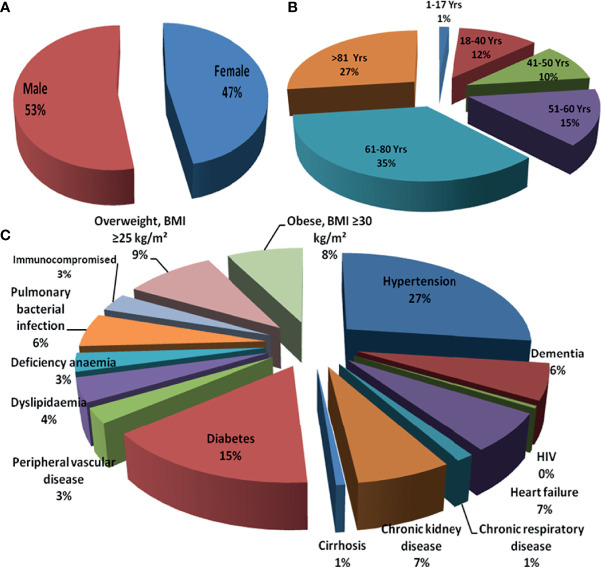
Risk of infectivity and morbidity among COVID-19 patients: **(A)** sex based, **(B)** age based, and **(C)** comorbidities. COVID-19, coronavirus disease 2019.

The case fatality rate (CFR) in COVID-19 seems to be elevated than that of in seasonal influenza, whereas both diseases principally have an effect on older adults above 65 years of age with infirmity (Dan et al., 2020; Yadav et al., 2021b). The augmented fatality rate of COVID-19 could be because of variations in underlying comorbidities of patients, pathogenicity of the causative agent SARS-CoV-2, immunity of population, and responses of host to the infection (Jha et al., 2020; Upadhyay et al., 2020a; Upadhyay et al., 2020b). It has been reported that the COVID-19 patients were more frequently obese and suffered from diabetes, hypertension, and dyslipidemia than influenza patients; on the contrary, the influenza patients often had cardiac failure, chronic respiratory disease, cirrhosis, and anemia (Piroth et al., 2021). Patients admitted to care centers with new variant of SARS-CoV-2 more frequently experienced acute respiratory failure, pulmonary embolism, septic shock, or hemorrhagic stroke, but less frequently developed myocardial infarction or atrial fibrillation (Dan et al., 2020). In-hospital mortality was comparatively multifold higher in patients with COVID-19 than conventional influenza patients (16.9% *vs.* 5.8%, respectively), with a relative risk of death of 2.9 (West et al., 2021b). Quantitatively, there was less pediatric patients (<18 years) for COVID-19 than influenza among the patients admitted in the hospital, but a bigger proportion of patients younger than 5 years required intensive care unit (ICU) support to COVID-19 than influenza (Piroth et al., 2021). As per the report, in-hospital mortality of adolescents (11–17 years) was manyfold higher for COVID-19 than for influenza as well. Thus, the effect of the SARS-CoV-2 variant is tremendous for all sex and age groups of the human population but was supposed to be the most common challenging health risk factor to immunocompromised septuagenarians and octogenarians (**Figure 1**).

## Susceptibility of Coronavirus Disease 2019 to Clinically Used Drugs

Currently, the strategy to treat the COVID-19 infection comprises social distancing and vaccination. However, with the sharp rise in the cases and variable symptoms, various pharmacotherapies were explored for enhancing viral clearance and other symptomatic relief (Rahman and Idid, 2021). Until now, no specific drug for the treatment and management of COVID-19 has been developed. Hence, the focus has been shifted towards drug repurposing, which is time saving, is an accepted approach, and has an unmet need of time (Stasi et al., 2020). At present, many of the existing drugs have been repurposed and tested in preclinical and clinical trials (**Table 2**). However, with the advancement and better understanding of pathophysiology and clinical presentation among patients, it was noticed that the clinical efficacy of these drugs depends on timing of use, disease stage, and dose regimen (Iqubal et al., 2021a). Antiviral drugs are important when used during the early stage, as they inhibit viral entry and replication (Şimşek Yavuz and Ünal, 2020). Among antiviral drugs, remdesivir is one of the extensively used drugs. Initially, the *in vitro* study has shown antiviral potential against COVID-19. Later on, the US Food and Drug Administration (FDA) approved this drug to shorten the recovery time in adults and children (below the age of 12) (Young et al., 2021). However, the outcome of the WHO SOLIDARITY trial that involved 11,330 patients across 40 countries showed a non-significant effect on reducing mortality, duration of hospitalization, and need of a mechanical ventilator (Horby et al., 2020). Lopinavir/ritonavir is a combination therapy for HIV, and it was proposed to be an effective therapy for COVID-19 (Cao et al., 2020). Ivermectin is an approved antiparasitic drug (Caly et al., 2020). Initially, the *in vitro* study showed that ivermectin significantly inhibited the replication; but based on the outcome of a double-blinded randomized trial, no clinical efficacy of lopinavir/ritonavir and ivermectin among COVID-19-infected patients were found (López-Medina et al., 2021). These drugs are not in use now. Hydroxychloroquine and chloroquine were also claimed to be promising therapeutic modality against COVID-19 infection, but the outcome of the randomized trial showed a non-significant effect against symptomatic relief among COVID-19 patients (Mitjà et al., 2021).

**Table 2:** Details of various repurposed drugs in COVID-19 infection.

Class of drugs		Drugs	Mechanism of action	References
**Antivirals**		Remdesivir	Inhibitor of RNA-dependent RNA polymerase and, hence, compete for viral ATP, which results in inhibition of viral replication	Young et al., 2021
		Lopinavir/ritonavir	Inhibitor of 3-chymotrypsin-like protease (3CL^pro^) and inhibit viral replication	Cao et al., 2020
		Ivermectin	Blocker importin α/β receptor and, hence, inhibit the transmission of viral protein into the nucleus of host cell	Caly et al., 2020
		Ribavirin	Potent inhibitor of viral RNA synthesis	Iqubal et al., 2021b
		Favipiravir	Inhibitor of RNA-dependent RNA polymerase and, hence, compete for viral ATP, which results in inhibition of viral replication	Iqubal et al., 2021b
		Umifenovir	Affects the S protein activity and, hence, inhibit its fusion with the host cell	Iqubal et al., 2021b
**Immunomodulators**		**Corticosteroids** Dexamethasone Hydrocortisone Methylprednisolone	Effectively mitigate the pro-inflammatory signaling pathways, stimulate the anti-inflammatory pathways, inhibit COX as well as NF-kB-mediated hyperinflammation, and, hence, reduce the cytokine storm	Hamilton et al., 2021
		**IFN β-1a**	Potentiate the interferon and assist in viral clearance	Davoudi-Monfared et al., 2020
		**IL-6R-antagonists** Tocilizumab Sarilumab	Inhibit IL-6-mediated hyperinflammation and cytokine storm	Michot et al., 2020; Gordon et al., 2021
		**IL-1R antagonists** Anakinra		
		**TNF-α inhibitors** Adalimumab	Inhibit TNF-α-mediated hyperinflammation and control cytokine storm	Iqubal et al., 2021a
		**Bruton’s tyrosine kinase inhibitors** Ibrutinib Rilzabrutinib Acalabrutinib	Potent inhibitor of TLR-4 activation and, therefore, mitigate the cytokine storm and inflammatory pathway	Roschewski et al., 2020
		**JAK inhibitors** Baricitinib Fedratinib	Inhibit JAK and activate STAT pathway, leading to inhibition of cytokine production and maturation. Additionally, these drugs inhibit the viral endocytosis *via* interacting with ACE2	Stebbing et al., 2020
		**Calcineurin inhibitors** * Cyclosporine * Tacrolimus	Reduced the production of T-lymphocytes *via* tumbling the expression of IL-2 receptor and production of IL-2. Inhibit the viral replication	Cavagna et al., 2020
**Complement inhibitors**		Eculizumab	Inhibit the production of inflammatory C5a and C5b-9	Laurence et al., 2020
**Kinin–kallikrein pathway inhibitors**		Lanadelumab	Inhibitor of kallikrein and hence offers relief from ARDS	Lipcsey et al., 2021
		Icatibant	Antagonist of bradykinin receptor type 2 and thus, inhibit hyperinflammation	
**Serine protease inhibitors**		**C1 esterase inhibitor** Camostat mesylate Nafamostat mesylate	Inhibit the coagulation and ARDS *via* interacting with FXIIa and kallikrein	Urwyler et al., 2020
**Antimalarials**		Hydroxychloroquine	Inhibit the viral entry, replication, cytokine production and coagulation	Mitjà et al., 2021
		Chloroquine
**Blood-derived products**		Convalescent plasma	Maintain and stimulate the physiological defense against viral infection	Iqubal et al., 2021c
		Hyperimmune immunoglobulin
		Bamlanivimab	Anti-spike neutralizing IgG1 monoclonal antibody that interferes with the function of viral spike proteins	Gottlieb et al., 2021
		**REGN-COV2** Casirivimab Bamlanivimab Imdevimab Etesevimab Sotrovimab	Cocktail of two anti-spike neutralizing antibodies that that interfere the function of viral spike proteins	Tardif et al., 2021
**Miscellaneous**	Colchicine		Reduce hyperinflammation	Tardif et al., 2021
	Vitamin D		Maintain the immune function (innate and adaptive immune system). Reduce oxidative stress, inflammation and scavenge free radicals.	Giannini et al., 2021
	Azithromycin		Assist in viral clearance and inhibit viral replication.	Oldenburg and Doan, 2020
	Sirolimus		Inhibit T-cell differentiation *via* inhibiting mTOR pathway and, hence, reduces cytokine storm and ARDS.	Omarjee et al., 2020
	Bevacizumab		Inhibition of IL-6 and hence reduces the severity of cytokine storm and ARDS	Pang et al., 2021

COVID-19, coronavirus disease 2019; ARDS, acute respiratory distress syndrome.

Use of corticosteroids and immunotherapy is preferred during cytokine storms or at the hyperinflammatory stage, and inappropriate use of these drugs often results in fetal immunogenic reactions (Esmaeilzadeh and Elahi, 2021; Rabaan et al., 2021).

**Figure 2:**
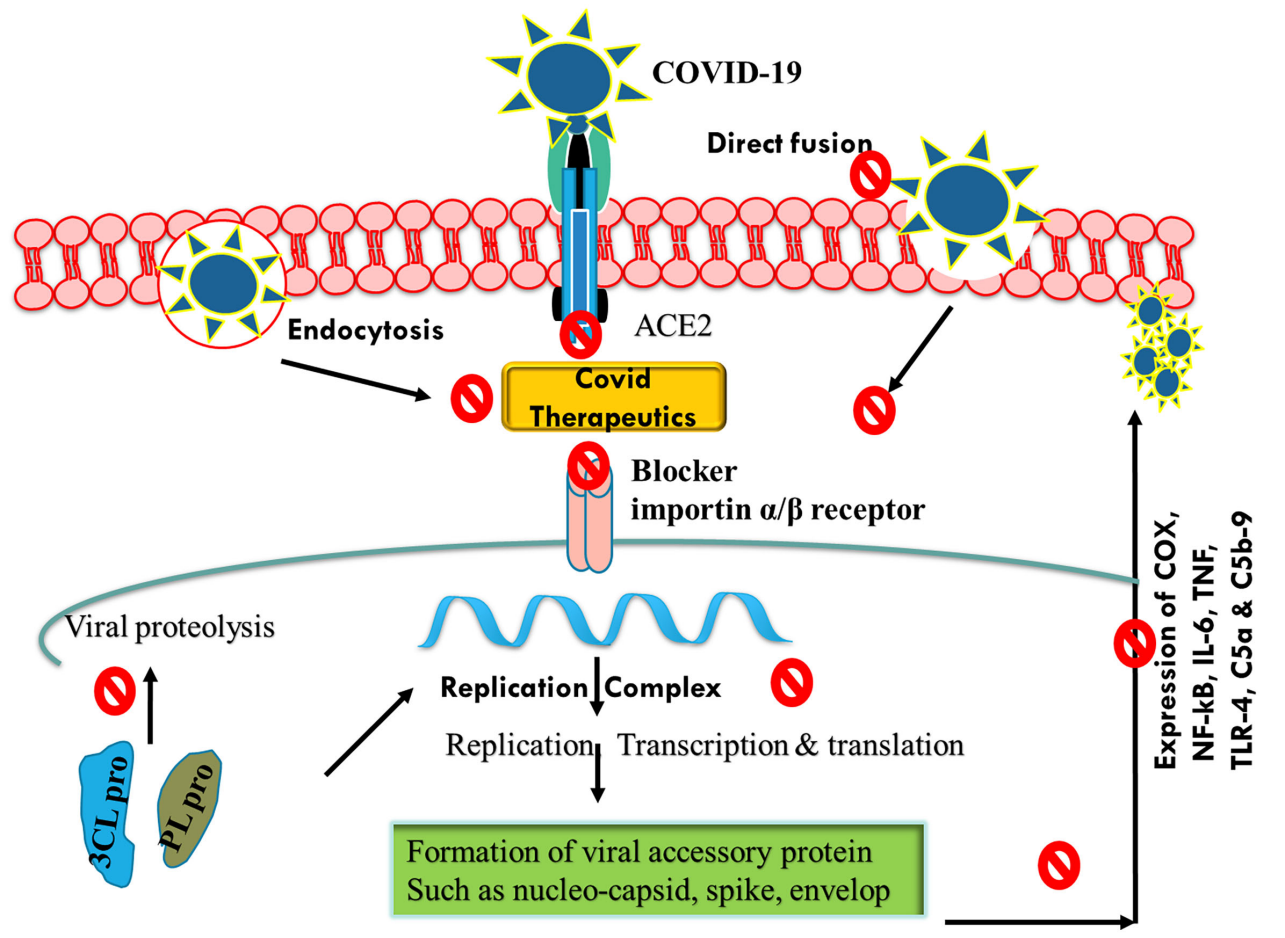
Schematic representation of mechanisms of action of COVID-19 therapeutics by inhibiting endocytosis, ACE2 receptor, and viral replication. COVID-19, coronavirus disease 2019; ACE2, angiotensin-converting enzyme 2.

Based on various clinical findings, corticosteroids were reported to be effective against cytokine storm and hyperinflated lungs (Hassan et al., 2020; Shang et al., 2020). The outcome of the landmark RECOVERY trial that involved confirmed patients of COVID-19 showed that the use of dexamethasone resulted in reduced mortality and need of mechanical ventilators or oxygen supply (Hamilton et al., 2021). Based on this trial, dexamethasone was approved among critically ill patients, either alone or in combination with remdesivir (Vetter et al., 2020; Mehta et al., 2021). Interferon-β-1a, a cytokine, exhibits an immunogenic response against viral infection (Yuen et al., 2020). Previously, interferon-β-1a showed clinical ineffectiveness against ARDS but exhibited a positive response among the patients of COVID-19 (Bosi et al., 2020; Kali et al., 2021; Tortajada et al., 2021). Interferon-β-1a, when used during the early stage of infection, reduced the duration of hospitalization and mortality rate (Davoudi-Monfared et al., 2020). However, recent findings have shown that interferon-β-1a is ineffective against Alpha (B.1.1.7), Beta (B.1.351), Gamma (P1), and Delta (B.1.617.2) strains (Davoudi-Monfared et al., 2020). Currently, interferon-β-1a is not recommended for treating COVID-19 patients (Davoudi-Monfared et al., 2020). Similar to interferon-β-1a, anakinra (interleukin-1 antagonist) was found to be effective in reducing mortality during the initial investigation, but recent findings have shown its ineffectiveness against B.1.1.7; B.1.351, and P.1 variants and, hence, are not recommended to treat COVID-19-infected patients (Huet et al., 2020). Tocilizumab (IL-6 receptor antibody) and sarilumab as well as siltuximab (IL-receptor antagonist) are effective during hyperinflammatory state; and hence, they were explored for possible protective effects in COVID-19 infection (Michot et al., 2020). Some clinical trials, such as REMAP and RECOVERY, showed the benefit of using tocilizumab, sarilumab, and siltuximab, which reduced mortality and showed a better safety profile among infected patients (Michot et al., 2020; Gordon et al., 2021). Janus kinase (JAK) inhibitors (baricitinib, ruxolitinib, and tofacitinib) are well-known drugs approved for rheumatoid arthritis and other inflammatory conditions (Stebbing et al., 2020). Baricitinib is considered as one of the potential drug candidates against COVID-19 infection (Saber-Ayad et al., 2021). This drug acts by inhibiting viral endocytosis in the *in vitro* study and inhibits the altered hyperinflammatory signaling pathway (Richardson et al., 2020). In ACTT-2 trial, when baricitinib was used in combination with remdesivir, it showed superior clinical efficacy in reducing ARDS and mortality rate as compared with baricitinib alone (Kalil et al., 2021). Currently, the combination of baricitinib and remdesivir is approved by the US FDA for the treatment of COVID-19 infection (Kalil et al., 2021). Apart from JAK inhibitor, Bruton’s tyrosine kinase inhibitors such as rilzabrutinib, ibrutinib, and acalabrutinib are currently approved by the US FDA for the treatment of hematological malignancy (**Table 2**) (Rezaei et al., 2020; Benner and Carson, 2021; Rada et al., 2021). These drugs act as an inhibitor macrophage activation, which is a rate-limiting step during cytokine storm (Roschewski et al., 2020). Therefore, these drugs are hypothesized to be a future therapeutic candidate against COVID-19 infection (**Figure 2**). More recently, anti-SARS-CoV-2-neutralizing antibodies such as casirivimab, bamlanivimab, imdevimab, etesevimab, and sotrovimab were approved by the US FDA for the treatment of non-hospitalized patients with a confirmed report of COVID-19 infection (Mahase, 2021; Verderese et al., 2021; Weinreich et al., 2021).

## Differences in Efficacy of Vaccines on Preventing Infection With Coronavirus Disease 2019 Strains and Controlling Necessity for Hospitalization

**Figure 3:**
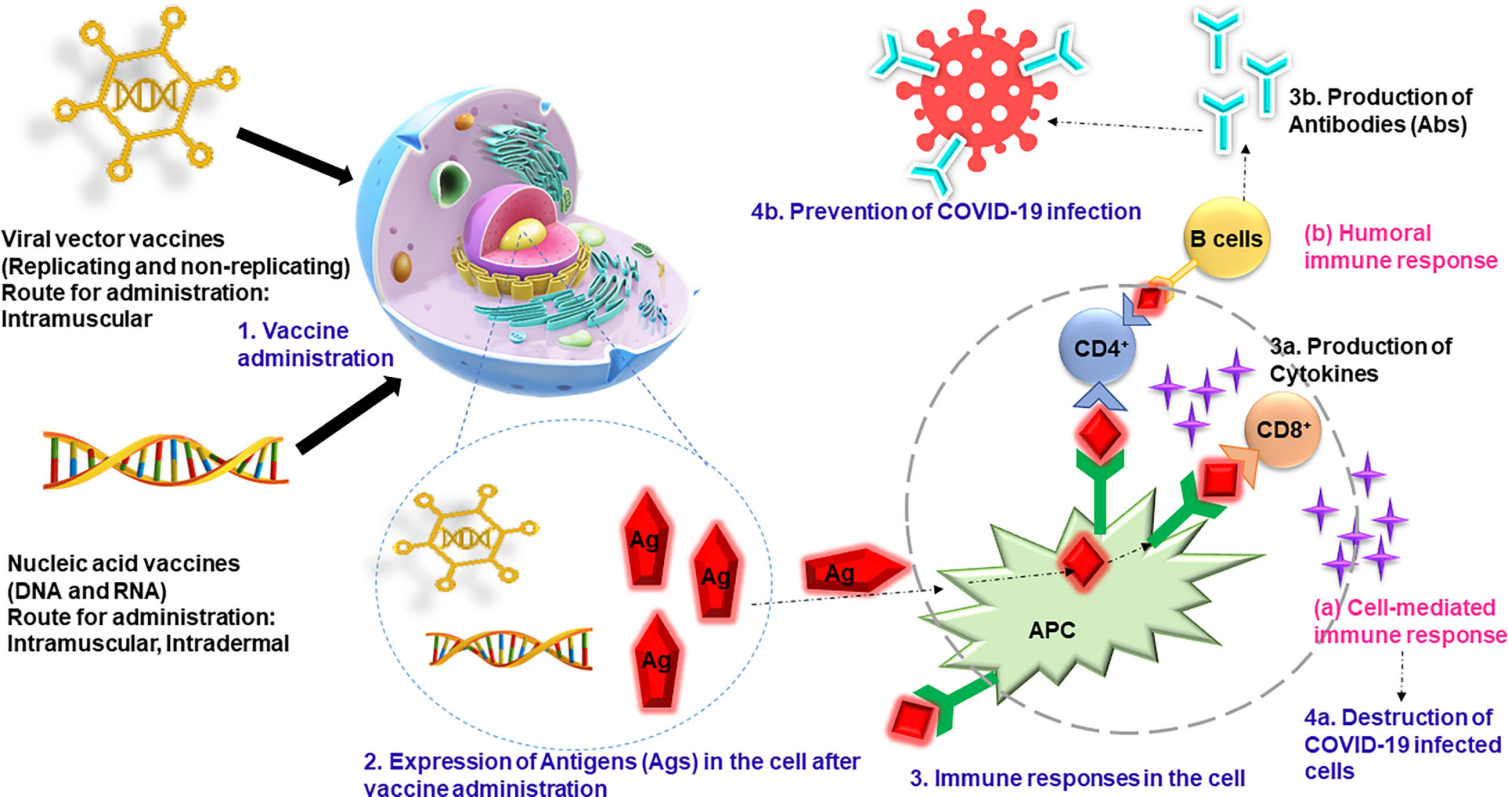
The vaccines (viral vector and nucleic acid vaccines) are administered through intramuscular or intradermal routes, and antigen expression is initiated in the cells. The B cells and T cells generate the humoral immune response and cell-mediated immune response, respectively. The cell-mediated immune response produces the cytokines that kill the infected cells, and humoral cells produce antibodies that prevent the COVID-19 infection. COVID-19, coronavirus disease 2019.

COVID-19 vaccines play a critical role in helping the countries to overcome the challenging pandemic that they are currently grappling with. It is believed that the severity of the pandemic will gradually reduce as the herd immunity is achieved. However, there may be factors that make it difficult to achieve herd immunity such as receiving only one dose of the vaccine for which two doses are required, denial to get vaccinated, and shortage of the vaccines. Therefore, it is very important to mass vaccinate the population completely if we want to win the battle over the pandemic (Chen and Lu, 2021). A public–private partnership was initiated by the US government to speed up development, approval, and distribution of the COVID-19 vaccines (Corey et al., 2020). Most of the COVID-19 vaccines have spike glycoprotein of SARS-CoV-2 as their basis. The commonly used vaccines are as follows: BNT162b2 (Pfizer-BioNTech) (Polack et al., 2020), ChAdOx1 nCOV19 (Oxford-AstraZeneca) (Voysey et al., 2021), NVX-CoV2373 (Novavax) (Keech et al., 2020), mRNA-1273 (NIAID-Moderna) (Baden et al., 2021), and Ad26COV2S (Janssen) (Sadoff et al., 2021). There are several preprints, peer-reviewed publications, press releases, policy documents, and public regulatory documents that demonstrate the efficacy and safety of these vaccines (Keech et al., 2020; Polack et al., 2020; Baden et al., 2021; Voysey et al., 2021). A study was conducted to study the efficacy of BNT162b2 vaccine (Dagan et al., 2021) during the mass vaccination in Israel. The participants were followed up 7 days after the second dose, and it was found that the vaccine has an efficacy of 94% for symptomatic COVID-19 participants, 92% for people with severe COVID-19, 92% for people with documented infection, and 87% for the people admitted in the hospitals. It was also concluded that the effectiveness of the vaccine was lower in people who suffer from various coexisting medical conditions like hypertension and obesity than in healthy individuals. Similar results were found in England for adults aged 70 years and over, indicating that the BNT162b2 vaccine showed 85%–90% efficacy after the second dose (Lopez Bernal et al., 2021). The risk of being admitted to hospitals was reduced by 44% in the vaccinated people, whereas the risk of death was reduced by 51%. They also studied the efficacy of ChAdOx1-S vaccine and found out that a single dose was 60%–75% effective in people with symptomatic COVID-19 and that the risks of hospital admission were reduced up to 80% in the vaccinated people. Various vaccines are being manufactured and distributed across the globe (**Table 3**) to control the pandemic. **Figure 3** summarizes the mechanisms of action of investigated anti-COVID-19 vaccines. These vaccines have helped in reducing the number of COVID-19 cases; however, the efficacy may vary in different studies. In the earlier phases of vaccination, it was found that the people receiving the vaccination were more prone to COVID-19 infection, which encouraged people to defer the vaccines. However, it was found that the infection occurred when people travelled to infected region or encountered COVID-19-positive patients, and the risk of infection was higher in the first 3 days of vaccination. This period was before the incubation of vaccine occurred, which rules out the odds of vaccination.

**Table 3:** Various vaccines available for COVID-19.

Vaccine	Manufacturer	Origin of vaccine	Dose(s) required	Efficacy against COVID-19
BNT162b2 or Comirnaty	Pfizer-BioNTech; Fosun Pharma	m-RNA-based vaccine	2 doses, 21 days apart	94% (Dagan et al., 2021)
ChAdOx1-S or AstraZeneca or Covishield (India)	Oxford-AstraZeneca	Adenovirus vector expressed in chimpanzee	2 doses, 28 days apart	60%–75% (Lopez Bernal et al., 2021)
NVX-CoV2373	Novavax	Spike protein expressed in baculovirus	2 doses, 21 days apart	95.6% (Mahase, 2021)
Gam-Covid-Vac or Sputnik V	Gamaleya Research Institute, Acellena Contract Drug Research and Development	Spike protein expressed in adenovirus Ad5 and Ad26 vectors	2 doses, 21 days apart	92% (Roxby, 2020)
Moderna COVID-19 vaccine or mRNA-1273	Moderna, U.S. Biomedical Advanced Research and Development Authority (Dagan et al., 2021), National Institute of Allergy and Infectious Diseases (NIAID)	m-RNA vaccine expressing adenovirus type 26 (dose 1) and adenovirus type 5 (dose 2)	2 doses, 28 days apart	94.5% (Voysey et al., 2021)
Covaxin	Bharat Biotech, Indian Council of Medical Research (ICMR)	Inactivated virus vaccine	2 doses, 28 days apart	81% (Biotech, 2021)
BBIBP-CorV	Beijing Institute of Biological Products; China National Pharmaceutical Group (Sinopharm)	Inactivated virus vaccine	2 doses, 21 or 28 days apart	79% (Yan et al., 2021)
JNJ-78436735 or Ad26.COV2.S or Janssen COVID-19 vaccine	Janssen Biotech Inc.—Janssen Pharmaceutical Company of Johnson & Johnson	Spike protein expressed in adenovirus Ad26 vector	2 doses, 56 days apart	76.7%–85.4% for severe COVID-19 patients (Yan et al., 2021)
CoronaVac	Sinovac	Whole inactivated virus vaccines with alum as an adjuvant	2 doses, 14–28 days apart	50%–91% (Yan et al., 2021)
EpiVacCorona	Federal Budgetary Research Institution State Research Center of Virology and Biotechnology	Subunit vaccine	2 doses, 21–28 days apart	100% (Phase I and Phase II trials) Merah et al. (2021)
Ad5-nCoV or Convidicea	CanSino Biologics	Spike protein expressed in adenovirus Ad5 vector	1 dose	90.98% (interim analysis) (Peshimam and Farooq, 2021)
ZF2001	Anhui Zhifei Longcom Biopharmaceutical, Institute of Microbiology of the Chinese Academy of Sciences	Recombinant vaccine	3 doses within 90 days	NA
Name not yet announced	Wuhan Institute of Biological Products; China National Pharmaceutical Group (Sinopharm)	Inactivated vaccine	NA	72.5% (interim analysis) (Yan et al., 2021)

COVID-19, coronavirus disease 2019.

NA, Not Applicable.

Most of these vaccines were manufactured against the original strain of SARS-CoV-2, and since then, the virus has mutated several times. It is crucial to develop a wide-spectrum vaccine that is effective against the various strains of SARS-CoV-2. In addition, for controlling the COVID-19, it is very important that the global population may be vaccinated completely. It is the duty of the officials to build trust among the public and encourage them to get vaccinated. The eradication of this disease is only possible when the herd immunity is achieved by vaccinating the people globally.

## Possibilities to Facilitate Overcoming Coronavirus Disease 2019 Pandemic

Considering the rapid molecular evolution of SARS-CoV-2 virus from its emergence to the present moment, continuous surveillance is required to identify novel mutations with potential ability to bypass current measures for controlling COVID-19. In the near future, readiness to react to such changes in virus genome is probably unavoidable. Rapid ongoing vaccination with continuously improved and updated vaccines or even vaccine cocktails is obviously the only human-controlled proactive way to impede the pandemic. Taking into consideration the fact that increased transmission can enhance the probability of further mutations (Matta et al., 2021), quick vaccination of the most active (younger) age groups seems to be the best strategy for preventing the appearance of novel hazardous mutations. On the other hand, the possibility of emergence of a mutant virus variant with high prevalence (high transmissibility) but low virulence cannot be avoided, overriding the spread of the current high-lethality strains and changing the fatal disease course to be much milder, thereby ensuring the “friendly” coexistence of virus and humankind in the future. Which of these scenarios will come true is just the question of time; still, it is clear that the lessons that this pandemic has taught to humankind are absolutely unique and tremendous.

## Conclusions

Within already nearly the last 2 years, humankind of the 21st century has undergone unexpectedly complicated challenges related to the COVID-19 pandemic, from total social isolation to different mass-vaccination campaigns. However, despite biotechnological prosperity and ultrafast preparation of vaccines, we still cannot look to the future with peace of mind, as the virus is circulating among populations even after the use of current vaccines, and we have no means to forecast the virulence and lethality of potentially developing novel strains. Therefore, our location within this pandemic can be decided only retrospectively, and it remains to be hoped that after 5 years we will estimate today’s position as the end of the pandemic.

## Author Contributions

HT performed the literature survey and data extraction. KS contributed in the introduction and conclusion. PA contributed in the molecular evolution. AI contributed in the therapeutic section. SU contributed in the geographic distribution section. JK contributed in the vaccination section. GK and DA contributed in final proofing and editing. All authors contributed to the article and approved the submitted version.

## Conflict of Interest

The authors declare that the research was conducted in the absence of any commercial or financial relationships that could be construed as a potential conflict of interest.

## Publisher’s Note

All claims expressed in this article are solely those of the authors and do not necessarily represent those of their affiliated organizations, or those of the publisher, the editors and the reviewers. Any product that may be evaluated in this article, or claim that may be made by its manufacturer, is not guaranteed or endorsed by the publisher.
